# Letter from London

**Published:** 1881-12

**Authors:** W. T. Belfield

**Affiliations:** London


LONDON, Nov. 6, 1881.

EDITORS CHICAGO MEDICAL JOURNAL AND EXAMINER:—The medical visitor in London is impressed during his progress through the city in almost any direction by the immense numbers and diverse purposes of the hospitals. One would infer that almost every cubic inch of the human form divine was the object of special study and treatment. The chest, throat, eye, ear, genito-urinary tract, rectum, brain, teeth, skin, hip-joint, have each one or more hospitals professing to treat the special diseases to which the organ in question is subject; beside which children, women pregnant and women not pregnant, consumptives, cancerous patients, the paralyzed and epileptic, the insane and those suspected of a clandestine tendency toward insanity, the syphilitic, the fever patients, those requiring orthopædic treatment, are the objects of tender solicitude to hospitals treating these various ailments respectively. Besides these are the numerous general hospitals, and almost innumerable dispensaries, public and private.

Yet all these various institutions have, with the exception of a few well-endowed hospitals such as Guy's and St. Bartholomew's, one phase in common, namely, that they are "supported by voluntary contributions," a fact published in enormous letters in a most conspicuous place on the building. A series of boxes placed at salient points and adorned with scriptural quotations, with touching allusions to your deceased aunt, explains itself. Yet most of these institutions are more or less endowed, and not strictly dependent upon the benevolence of the public.

Another more agreeable feature is also common to all. With rare exception, the simple presentation of one's professional card secures him the courteous attention of the physician in attendance, and a cordial invitation, at the conclusion of the visit, to come again.

The natural inference, from the sight of these hospitals, that the London profession is an army of specialists, is but half correct. It is true that most of the better men study especially a particular organ or system of organs, but not to the exclusion of the rest. The London ophthalmologist is a general surgeon, the London neurologist is a general physician. Hence it happens that the specialist often knows surprisingly little of his own "particular vanity," while the general physician or surgeon seems astonishingly familiar with various specialties. Yet no one, with rare exceptions, attempts to cover more than half the medical field—medicine proper, or surgery. The line of demarkation between physician and surgeon is sharply drawn in professional qualifications as well as in practice. The physician is supposed to have studied longer, and to have passed more trying ordeals of examination ; he affects somewhat contemptuous ignorance of matters surgical, and calls in a surgeon even to amputate a finger. The surgeon, on the other hand, while appropriating everything requiring manual interference, and doing "light jobs" on the viscera, refers with reverent humility all the more important internal lesions to the supposed superior wisdom of the physician. Nor is this distinction merely formal, as I was at first inclined to suppose. I once heard Mr. Hutchinson's assistant surgeon, in narrating Mr. H.'s endeavors to elucidate the relations between certain skin diseases and systemic conditions, say, by way of apology for lack of success, "Mr. Hutchinson is, however, only a surgeon." The man whose work is familiar to all is supposed to know nothing more about the body than the probe, scalpel and ophthalmoscope reveal ; and has no other conventional title than if he were a successful brewer or politician !

That Jonathan Hutchinson is so ignorant of medicine I do not believe ; yet a surgeon of a large hospital, upon being consulted the other day as to the (possibly aneurismal) nature of a tumor in the thigh, called for a stethoscope ; a binaural instrument was offered him ; he hesitated, then, with the firmness of despair, grasped it manfully, applied it to his ears, point backward, and after listening (?) a moment, gravely, and doubtless truthfully announced, that he heard nothing. An incision revealed the wall of an aneurism about one-half inch below the skin.

The medical teaching here presents great advantages combined with glaring deficiencies. I will abstain from discussion, and content myself with remarking that while the student has here opportunities for clinical observation unequaled at any school, American or Continental, of which I have personal knowledge, there is, nevertheless, such imperfect individual instruction in methods of diagnosis as to permit a man to remain quite unacquainted with microscope, ophthalmoscope, laryngoscope, and even stethoscope. The students in a certain large hospital here call all adventitious sounds in the lungs "crepitations." A careful study as to what a "crepitation" was, revealed to me, under that name, sometimes the moist râles of bronchitis, sometimes the dry rales of asthma, again the crepitant râle of incipient pneumonia, but especially the gurgling râles of advanced phthisis. Whether any distinction is made as to the pathological significance of these various "crepitations," I am unable to say.

A word as to hospital management, in discussing which I shall refer to the London Hospital—the largest though not the wealthiest in Great Britain—where I have for four months been clinical assistant.

Aside from the usual amount of red tape essential to the average Briton's ideas of propriety here and hereafter, the management is generally excellent. The board of managers or "governors" (this word is doubtless chosen to imply, not a judicial, but a paternal relation to the hospital, since it is young Britannia's pet name for his most immediate male ancestor), is less obnoxious than such bodies usually are. The domestic affairs are in the hands of non-professional men, but are probably as good as possible under these circumstances. But it is of the ward work that I wish especially to speak. Each ward, containing ordinarily about forty-five beds, is furnished with a head-nurse, technically called "sister," and two assistants—all of them of better social grade than the usual American or Continental nurse; indeed it is not uncommon to see ladies (in the English sense) performing these onerous duties. These three are replaced at night by a fourth nurse. The sister is responsible for the details of the nursing, and performs, personally, the less disagreeable work. Every medical ward is provided with drugs required for emergencies; a set of tubes, reagents, etc., for urinary examinations; a laryngoscope, stethoscope, hypodermic syringe. The surgical wards have each a small operating case, air-cushion dressings, etc. Ventilation is by flues, and dubious. Iron bedsteads with hair-mattresses are used, the bed provided with half curtains, which secure to the patient a certain degree of privacy, at least above the waist. Over each bed hang two written records—one containing directions for the nurses—medicines, applications, etc.; the other a history of the case; a chart of the temperature, taken twice daily; the quantity of urine passed in ounces; the number of stools (all recorded by the nurses), and the condition of the urine, recorded once a week by the clinical clerk. Diagrams are provided, showing in outline the various regions of the chest and abdomen, for convenience and accuracy in recording the location of tumors, lesions, etc.; in special cases charts for electrical reactions, elaborate analyses of urine in diabetes, appearances of the fundus oculi in cranial disease, etc., are furnished.

In a wire frame hang cards naming the articles which compose the patient's diet. Stimulants—*i. e.*, distilled—are used only in special cases, but beer is included among dietary articles.

The floors are plain wood, a broad matting extending down the center of the ward. A comfortable fire on a large hearth relieves the otherwise desolate aspect. The mental and moral needs are supplied by a profusion of pious texts distributed around the walls; by prayers from the Episcopal service at each bed, and by a few dozen books.

Children are accommodated in four large wards; and two others are devoted to Hebrew patients. No maternity nor contagious fever cases are admitted.

The points of interest in the practice of this and other hospitals will be referred to later. W. T. BELFIELD.

